# Derotational Subtrochanteric Osteotomy and External Fixation for the Treatment of Neurogenic Hip Dislocation in Children with Cerebral Palsy: Could This Be a Viable Method of Treatment?

**DOI:** 10.7759/cureus.7437

**Published:** 2020-03-27

**Authors:** Stavros Angelis, Georgios Vynichakis, Angelos Trellopoulos, Alexandros Apostolopoulos, Dimitrios Filippou, Marios Salmas, Michail Chandrinos, Theodore Balfousias, Leonidas Palaiodimos, Niki Kyriazi, John Michelarakis

**Affiliations:** 1 Surgical Anatomy, National and Kapodistrian University of Athens Medical School, Athens, GRC; 2 Orthopaedics, Panagiotis & Aglaia Kyriakou Children's Hospital, Athens, GRC; 3 Orthopaedics, Korgialenio-Benakio Hellenic Red Cross Hospital, Athens, GRC; 4 Orthopaedics, General Hospital of Piraeus Tzaneio, Piraeus, GRC; 5 Orthopaedics, Hygeia Hospital, Athens, GRC; 6 Orthopaedics, East Surrey Hospital/Surrey and Sussex Healthcare National Health Service Trust, Redhill, GBR; 7 Surgery, National and Kapodistrian University of Athens Medical School, Athens, GRC; 8 Orthopaedics, National and Kapodistrian University of Athens Medical School, Athens, GRC; 9 Internal Medicine, Montefiore Medical Center/Albert Einstein College of Medicine, New York, USA

**Keywords:** cerebral palsy, subtrochanteric osteotomy, external osteosynthesis, swivelling clamp, dislocated hips, reimers’s migration index, tönnis classification system, gross motor function classification scale, shenton’s line

## Abstract

Purpose

The treatment of painful and chronic dislocated hip in children with severe cerebral palsy (CP) is particularly demanding and controversial. Numerous surgical techniques have been described, and their outcomes vary a lot. The purpose of the present study is to evaluate a new method, which combines varus derotational subtrochanteric osteotomy (VDSO) and external osteosynthesis: (VDSOEO).

Methods

Six non-ambulatory children with spastic quadriplegia and chronic dislocated painful hips were treated. The technique involved a small incision on the subtrochanteric site of the osteotomy, followed by retention with a single-sided external osteosynthesis with rotational correction capability [swiveling clamp (SC)] for the reduction of the femur head in the acetabulum, and finally by the osteotomy. Hardware was removed without a second intervention four-six months postoperatively and after the osteotomy was healed. Evaluation of the method was based on clinical, functional, and radiological criteria.

Results

Four patients achieved improved radiological scores. Two patients demonstrated resubluxation during the period of the osteotomy's healing process. However, no patients experienced pain, and all were able to sit post-surgery, while caregivers reported improved capacity for nursing care.

Conclusions

It is our strong belief that this approach can improve the quality of life in children with severe CP and painful and chronic dislocated hips. It is a viable and definitely less invasive procedure than classic pelvic or femur osteotomies.

## Introduction

Hip displacement in cerebral palsy (CP) patients is a common condition that pedoorthopaedic surgeons are called to address upon. There is no consensus on the prevalence of neurogenic hip dislocation among children. Studies indicate a wide range of incidence, ranging up to 90% [1].

CP is an upper motor neuron disease characterized by a combination of spasticity and muscle weakness [2]. Patients are born with normal hips [3]. Abnormal and deforming muscle forces around the hip joint lead to its dynamic deformity. The long-term tenacity of this condition may result in contractures, bony deformity, and, ultimately, joint subluxation/dislocation [1-5]. These deformations, which may also be accompanied by pain, have an adverse effect on patients' quality of life. Standing, positioning, sitting, hygiene, and general care measures are usually affected [3,4].

Preventive, reconstructive, or salvage procedures usually aim to offer a pain-free hip that allows for an adequate range of motion and contributes to a better quality of life [3,4,6]. Many operative procedures have been invented to address patient-specific problems relating to the condition. These procedures range from soft-tissue reconstructions and releases, femur or pelvic osteotomies and bony reconstructions to arthrodesis, femoral head resection, or arthroplasty [1,3-5]. Our study aims to evaluate the viability as well as the advantages and disadvantages of a new minimally invasive technique that combines varus derotational subtrochanteric osteotomy (VDSO) and external osteosynthesis: (VDSOEO).

## Materials and methods

Study design

This is a single-clinic retrospective review case series of all CP patients with hip dislocation treated by VDSOEO between January 1, 2017 to January 1, 2018. Medical records from these patients were queried after board approval with the follow-up taking place at a specialized outpatient clinic.

The preoperative and postoperative clinical and radiographic assessment included demographic data, classification of neurological deficit type, concomitant preceding procedures on the ipsilateral limb of interest, continuity of Shenton’s line, acetabulum condition, Gross Motor Function Classification Scale (GMFCS), Reimer's migration index (RM), and Tönnis classification system [2,7-9]. The pain was assessed qualitatively after being reported by the patient or family. Complications associated with the operation, number of surgeons who implemented the VDSOEO method, and time to the radiographic union (complete healing of all four cortices on anteroposterior and lateral radiographs) and removal of external osteosynthesis were also evaluated. The main goals following the operation and removal of the external osteosynthesis were to facilitate nursing care, alleviate pain, and allow sitting posture in a wheelchair or mobility from bed to chair.

Follow-up meetings were scheduled and carried out monthly for the first six months followed by every six months. Family members were educated on how to properly attend to the patients.

Surgical technique

All patients were operated under general anaesthesia. The patient was placed on a radiolucent table in a supine position. The ipsilateral arm was positioned across the chest so that it did not interfere with the area of interest. The involved lower extremity was prepared and draped and the hip was held in a neutral position. The femur was visualized with the X-ray image intensifier and the area just distal to the lesser trochanter (osteotomy site) was identified and marked.

The procedure resembles the technique described by Hefny et al. who used a combination of valgus osteotomy and external fixation for the treatment of developmental coxa vara [10]. The first step was to place the half-pins in the proximal segment with the limb in the hip-neutral position. This overcomes the need for extensive skin release around the half-pins following the corrective osteotomy. The proximal half-pin was placed in a direction from superolateral to inferomedial. The approach did not involve the “modified technique” described by Hefny et al. [10]. In some cases, two half-pins were used instead of one for better grip control.

The second step was to place two or three half-pins distally to the osteotomy site perpendicular to the anatomical axis of the femur. For better control of the femur fragments and in order to avoid uncontrolled displacement, a single-sided frame was then mounted and the connecting rods between proximal and distal half-pins were not tightened until the osteotomy was performed [10]. This constituted the third step.

The fourth step involved a 2-cm longitudinal incision that was executed at the level of the marked osteotomy site in the anterolateral subtrochanteric area. This was followed by a direct deep approach to the bone of the subtrochanteric area through subcutaneous tissue, fascia lata, and the vastus muscle, in line with the skin incision.

**Figure 1:**
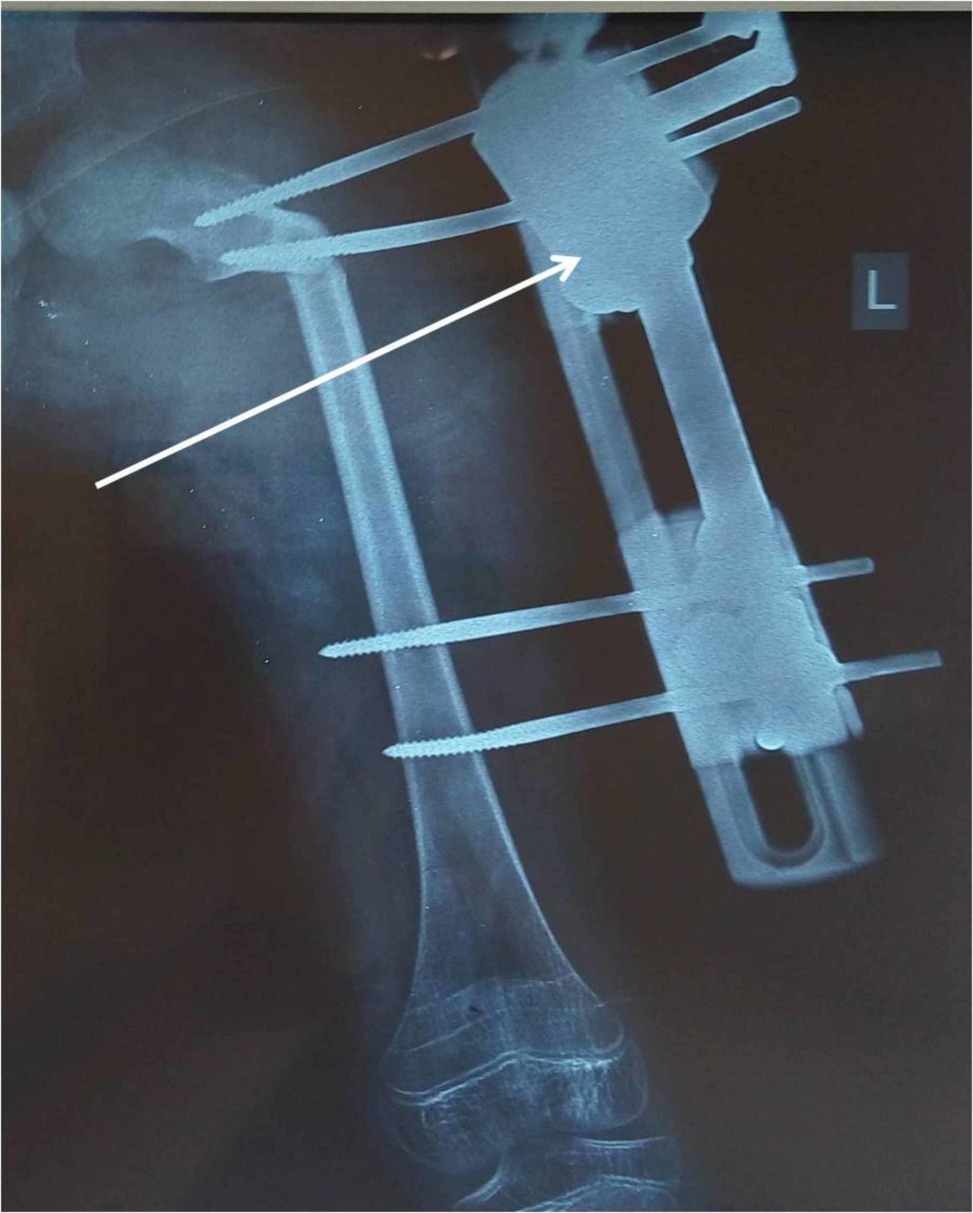
SC device (white arrow) implemented on the single-sided external osteosynthesis LRS The image shows the placement of two half-pins in the proximal segment for better grip control and two half-pins distally to the osteotomy site in a perpendicular angle with the anatomical axis of the femur SC: swiveling clamp; LRS: limb reconstruction system

Finally, osteotomy was performed by all possible techniques. Our approach involved the use of powered reciprocating bone cutters. The procedure was completed with osteotomes. Connecting rods were then tightened and the swiveling clamp (SC) was implemented on the single-sided external osteosynthesis limb reconstruction system (LRS) (Figure 1). Part of the correction was achieved during the surgery by tightening the SC system in a maximum improved varus position for the reduction of the femur head in the acetabulum. Additional correction may be progressively achieved for the first two weeks post-surgery by adjusting the SC system accordingly and as much as soft tissues allow.

## Results

VDSOEO was performed on six hip joints of six different patients (four females and two males). All six medical records were available for full analysis. The age of patients at the time of surgery ranged from 10 to 16 years with a mean age of 12.8 years. All suffered from the quadriplegic spastic form of CP.

Preoperatively, hip displacement in every patient was characterized as severe. None was ambulatory and four patients were unable to maintain a sitting posture. The dislocation was chronic and irreducible with dysplasia of the acetabulum, obvious disruption of Shenton's line, and a severe RM greater than 66% (mean RM: 91%) [5,8]. All patients were classified as level IV and V according to the GMFCS and as grade III or IV according to the Tönnis classification system [2,7,9]. The pain manifestation ranged from mild to severe (one patient reported mild pain, one moderate pain, and four patients or their family members reported severe pain). According to the concomitant preceding procedures record on the ipsilateral limb of interest, all but one patient had been subjected to some form of surgical intervention prior to VDSOEO.

VDSOEO in all patients was conducted by a single surgeon. The mean follow-up period was 1.75 years (range: one-three years). The mean time to union and removal of the external osteosynthesis system was five months (range: four-six months) (Table 1).

**Table 1:** Demographic data and functional and radiological scores of the sample ID: procedure number; GMFCS: Gross Motor Function Classification Scale; RM: Reimer's migration index; post: postoperative; pre: preoperative; F: female; M: male; D: disrupted; I: intact

ID	Time to union, months	Follow-up, years	Gender	Age at surgery, years	GMFCS	RM, %		Tönnis classification		Shenton’s line	
						Pre	Post	Pre	Post	Pre	Post
1	5	1	F	16	V	100	100	IV	IV	D	D
2	5	1,5	F	14	V	100	100	IV	IV	D	D
3	6	3	F	12	V	100	20	III	I	D	I
4	4	1	M	10	IV	66	30	III	II	D	I
5	5	2	F	12	V	80	30	III	II	D	I
6	5	2	M	13	IV	100	30	IV	II	D	I
Mean	5	1.75	-	12.8	-	91	51				

**Figure 2:**
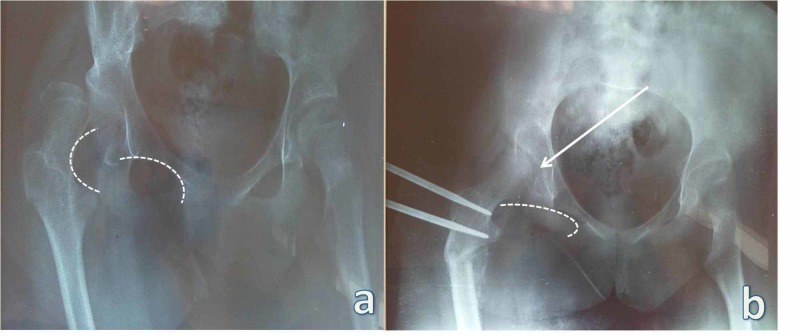
Restoration of femoral head-to-acetabulum congruity (arrow) a: X-ray demonstrating Shenton's line (interrupted curve) preoperatively; b: X-ray demonstrating Shenton's line (interrupted curve) postoperatively

Postoperatively and during follow-up, most functional and radiological scores demonstrated improvement. All patients were now able to sit in a wheelchair or able to move from bed to chair, while caregivers reported improved capacity for nursing care. Femoral head-to-acetabulum congruity and Shenton's line restoration were observed in four patients (Figure 2). In two patients, congruity and Shenton's line remained disrupted but had improved compared to the preoperative anteroposterior X-rays. Mean RM dropped to 51% postoperatively from a preoperative level of 91%. Tönnis classification system was improved by one or two grades in four patients. In the two patients who did not achieve congruity restoration, Tönnis classification grade remained the same as preoperatively. Finally, no patient or family member reported any pain one year after the surgery.

**Figure 3:**
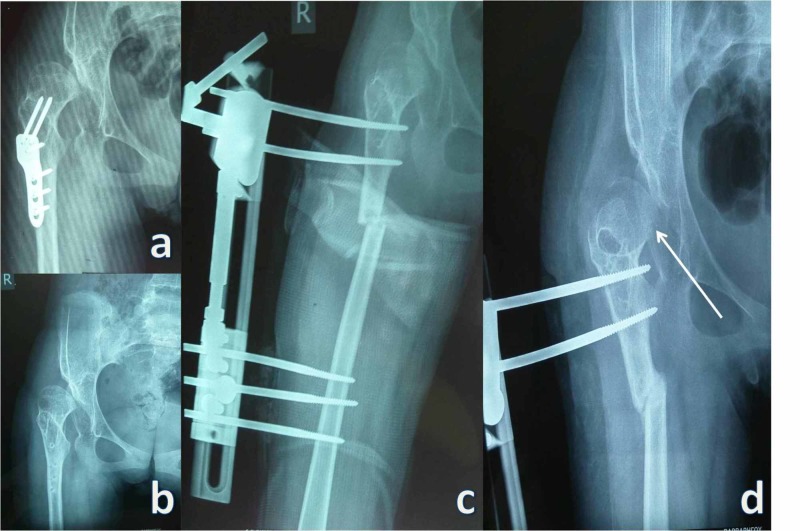
Pre- and postoperative hip X-rays of a patient a: preoperative hip X-ray of a patient who had undergone a failed osseous reconstructive procedure in the past; b: preoperative hip X-ray of the same patient after removal of the hardware of the first failed procedure; c: hip X-ray of the same patient demonstrating improvement of congruity and Shenton's line after the implementation of VDSOEO technique; d: hip X-ray of the same patient demonstrating resubluxation during the period of the healing process of the osteotomy (arrow). The image shows that the congruity and Shenton's line had improved compared to the preoperative anteroposterior X-rays VDSOEO: varus derotational subtrochanteric osteotomy and external osteosynthesis

Regarding postoperative complications, our first two patients demonstrated resubluxation during the period of the osteotomy's healing process. These patients had undergone other osseous reconstructive procedures in the past, and their hip deformity was fixed (Figure 3). Both patients were able to sit and experienced no pain one year postoperatively. No other important adverse effect was noted except for those related to half-pin care.

## Discussion

Hip displacement in CP patients is a common condition that may be prevented or treated on many occasions. Treatment is usually patient-specific and close monitoring of these patients is imperative for good outcomes. Treatment options include preventive, reconstructive, or salvage procedures when early conservative measures fail to provide a functional hip joint or at least improved quality of life [2,4,6]. As a sequela, when preventive and reconstructive procedures fail, or in neglected cases, salvage techniques may be recruited with the purpose to facilitate care for these patients. With this thought in mind, we implemented a simple and less invasive method that combines VDSO and external osteosynthesis in six non-ambulatory children with spastic quadriplegia and chronic dislocated hip who experienced mild to severe pain and problems with sitting posture and nursing care: VDSOEO.

VDSO is a well-established approach for treating CP dislocated hips [3,11,12]. The first report of this technique as a method of treatment for CP patients was described in 1959 by Phelps. However, in 1954, Jones was the first person to describe the use of femoral osteotomy for the treatment of dislocated hips in flaccid paralysis [13,14]. When VDSO is performed, the goal is to produce a stable painless concentric construction of the hip joint [3,11,15]. VDSO can be used as a single operation or in combination with other procedures. These may range from soft-tissue balancing to pelvic osteotomy and have been advocated by many researchers [15]. Others have suggested that severe preoperative subluxation/dislocation with a high RM is a risk factor that should be taken into consideration [3,16,17]. However, there is a number of studies that support the effectiveness of isolated VDSO without open reduction [3,11,18].

Until now, VDSO in CP was fixed under the use of internal osteosynthesis with angled blade plates or hip screws [19,20]. These procedures could have some drawbacks compared to the minimally invasive fixation technique with external osteosynthesis that we have proposed. Open procedures are prone to blood loss. Moreover, blood loss is more prominent when bone fragments from the osteotomy site have to be removed for the correct application of the plate [10]. Furthermore, bone removal may lead to further shortening of an already short extremity [10]. Another disadvantage of the internal fixation devices is that in many cases, they have to be secured on a limited percentage of bone in order to avoid the proximal femoral growth plate. This makes the construct precarious and open to instability and may require a hip spica cast for several weeks post-surgery [10]. Additionally, since the device is rigidly implanted into the underlying bone, adjustments and corrections are impossible [10]. However, our technique with the use of an SC device provides us with the ability to achieve additional varus correction during the first two weeks, making this procedure as adjustable as soft-tissue resistance allows. Finally, internal fixation devices need to be removed with another open procedure [10,21].

Our technique with the use of external fixation, on the other hand, may pose some disadvantages compared to internal fixation. From our experience, the most prominent patient complaint is that this bulky construction may be quite inconvenient. However, patient and family counseling may have good results [10]. Moreover, half-pin care is often challenging for the caregiver and should be discussed prior to surgery. Pin-tract infections can be a source of discomfort for the patient and his/her family, even if they are usually superficial. We should point out that the VDSOEO method was used as a salvage procedure for facilitating care and generally improving the quality of life for the patients.

Our results and analysis should be viewed in light of several limitations, the most important of which was the small number of patients. The second most important limitation was the retrospective study design and nonrandomized patient selection. All subjects participating in the study had demonstrated advanced functional and radiographic severity of hip dislocation prior to the operation. They experienced pain, with GMFCS levels of IV and V, Tönnis grades of III or IV, and RM greater than 66%. This would not allow us to suggest the technique for more moderate hip subluxations/dislocations since it was used only as a salvage procedure. We feel that these two limitations have prevented us from extracting definitive and more crosschecked conclusions. It is our strong belief that this method should be further investigated for its capabilities for prevention or reconstruction.

Despite these limitations, some important inferences have been drawn. VDSOEO is easily applied with a small learning curve and a minimally invasive technique that diminishes blood and bone loss as well as the shortening of an already short extremity. The procedure allows for accurate correction and rigid fixation, permits early mobilization, and avoids the need for a second operation for removal [10,22]. Additional varus can be applied during the first two weeks to maximize the correction that can be achieved. Finally, our data prove the effectiveness of the technique, endorsing our theory about the viability of the method in treating hip deformities in CP patients.

## Conclusions

VDSOEO with the use of an SC device is a new proposal for the treatment of hip subluxation/dislocation in CP patients. Our data support the effectiveness and viability of the method. We believe that the approach should be implemented on a larger scale in patients as a preventive, reconstructive, and salvage procedure for safer outcomes.
